# Supplementary material for: Are carbon emissions trading and green financial instruments synergistic? -Comprehensive quantitative research based on content analysis
Source: PLoS One. 2024 Mar 7;19(3):e0298601. doi: 10.1371/journal.pone.0298601 (PMC10919601; doi:10.1371/journal.pone.0298601)
Supplement: S1 Data — (ZIP) [file pone.0298601.s001.zip › Data/地级市Z_F工作报告.zip!/║■▒▒/╩«╤▀╩╨/└┤╫╘╣1⁄2╓┌║┼í╛┬φ┐╦╩2╛▌═°í┐_2014.docx]

各位代表：

　　现在，我代表市人民政府向大会作工作报告，请予审议，并请各位政协委员和其他列席会议的同志提出意见。

　　一、2013年工作回顾

　　2013年，是丰收之年。一年来，面对宏观经济持续加大的下行压力和严重干旱的自然灾害，在市委的坚强领导下，全市上下牢牢把握“竞进提质”总要求，紧紧围绕“四保六增”总目标，锐意进取，攻坚克难，各项工作有力有为有效，圆满完成了年初既定的各项目标任务，奋力开创了经济社会发展的新局面。预计全年实现地区生产总值1080亿元，增长13%；规模以上工业增加值490亿元，增长13.2%；全社会固定资产投资915亿元，增长30.2%；社会消费品零售总额482亿元，增长13.1％；地方公共财政预算收入73.53亿元，可比增长18.1％；外贸出口突破4亿美元，增长18%；实际利用外资1.56亿美元，增长18.6%；城镇居民可支配收入1.8万元，增长12.5%，农民人均纯收入5165元，增长13%；万元生产总值综合能耗下降4.5%。

　　（一）致力于调整结构，国民经济强劲发展

　　--农村经济增量增效。大力实施“四个一批”、“四百万”工程，完成农业增加值143亿元，增长5%。连续14年实现耕地总量动态平衡，粮油生产实现“十连增”。新增特色产业基地30万亩，完成农产品加工产值200亿元，增长40%；新增省级农业产业化经营龙头企业8家，新增无公害、绿色、有机食品品牌34个，新增湖北名牌17个、国家地理标志保护产品11个，“竹溪贡米”国家级农业标准化示范区通过验收。新增农民专业合作社236家、家庭农场34家。除险加固水库68座，完成小农水重点县项目12个，18.6万人饮水安全问题得到解决。秦巴山片区扶贫开发加快推进，新一轮城区农村扶贫开发启动实施，完成65个重点村整村建设任务，搬迁4020户，减少贫困人口9万人。

　　--工业经济快速增长。坚持服务东风公司与振兴地方工业并举，强力推进园区建设，工业经济速度、质效明显提升，完成规模以上工业企业总产值1650亿元，增长17%；汽车产业产值1120亿元，增长23%；新增规模以上工业企业304家。东风与沃尔沃合资重组快速推进，东风商用车动力总成一期、东风装备工业园焊装、东风零部件悬架弹簧、湖北大运汽车、三环工业园等项目竣工投产。建设工业园区10平方公里以上，“满园工程”成效显著，引进工业项目282个、到资135亿元。

　　--第三产业持续繁荣。促进旅游业加快发展，推进物流业快速成长，第三产业实现增加值380亿元，增长12%，新增限上企业300家。全年接待国内外游客2910万人次，实现旅游收入202亿元，分别增长24.7%和25.3%，十堰成为全国百强旅游城市。新增4A、3A级景区各3家，旅行社创A实现全覆盖，武当山被评为中国最美地质公园。华西国际农商城、中商城市广场、武商滨江量贩店等项目加速推进。十堰国际汽车商贸城一期、国药控股现代物流园竣工，林安物流园、昌升国际商贸城开工建设，“武当珍品汇”进入京津冀市场，信息化改造农家店200家。各项贷款余额731.5亿元，比年初增加153.1亿元，增长26.5%。

　　--项目投资扎实推进。千方百计拓展投资渠道，锲而不舍谋划、争取、引进项目，投资规模迅速扩张，项目建设成效显著，全社会固定资产投资增速保持全省前列。争取建设用地指标3.1万亩，增加18.6%。38个省级、263个市级重点项目全部开工建设，16类重点骨干工程强力推进，超额完成年度计划。武当山机场全面开工建设。武襄十城际铁路预可研报告编制完成。十白高速建成通车，谷竹、十房、郧十高速进展顺利。丹江口城区至土关垭、房县城关至军店、竹山城关至潘口、竹溪楚秦大道四条一级公路建成通车，两条环库公路完成路基42公里。汉江航道丹白段整治完工。孤山电站施工大桥建成，夹河关电站全部要件编制完成，龙背湾电站下闸蓄水，京能热电辅助工程开工。

　　（二）致力于统筹城乡，“一核多支点”格局加快形成

　　--中心城区发展和建设力度加大，“一核”地位得到巩固。城区经济增势良好，新建园区近万亩，新落户企业150家。张湾区引进过亿元项目25个，引资40.1亿元，增长90.6%；茅箭区大力发展服务业，实现增加值91.7亿元，对GDP的贡献率达60.8%；十堰经济开发区着力打造科技强区，实现高新技术产值298亿元，增长22.4%，占全市比重达71%。城市基础设施不断完善，规划建设管理水平明显提升。区域性中心城市发展建设规划、生态滨江新区控规及核心区城市设计规划编制完成。车城广场投入使用，中岳路、和谐大道、发展大道、机场大道西段、风神大道一期、林荫大道二号线一期、建设大道二期建成通车，郧阳路、京东路、西城大道、凤凰路、火车站北广场建设加快推进，改造5处交通堵点，完成350公里污水管网建设，天然气入户13.5万户。城中村、棚户区改造安置区13处，面积300万平方米。新增游园7个、绿地157万平方米、停车位1030个。生态滨江新区统一大道等4条道路竣工，天马大道等6条道路完成路基建设，新博物馆启动建设。“三违”治理拆除违法建筑85万平方米，处理小产权房73万平方米。持续巩固创卫成果，城市管理更加规范。

　　--县域发展提速增效，“多支点”特征初步显现。各县市因地制宜，明确定位，彰显特色，特色产业壮大，发展后劲增强。完成生产总值490亿元，增长13%；地方公共财政预算收入可比增长27.1%；实际利用外资增长25.8%；外贸出口增长22%；完成投资600亿元，增长30%，占全市比重达65.6%。丹江口润京水业、郧县郧府薯业、房县粤能生物质发电、郧西真武酒业、竹山沛丰粮油加工、竹溪梅子贡茶多酚等一批项目竣工投产。

　　（三）致力于改革开放，发展活力明显增强

　　--各项改革深入推进。加快建设多层次资本市场，再次成功发行16亿“城投债”，光大证券、中信银行、湖北银行入驻十堰，6家企业分别在武汉股权托管中心、天交所挂牌上市，安远专汽、东风减震器进军“新三板”市场，连续八年获得全省“金融信用市”殊荣。在全省率先启动新农合大病医疗保险，县级公立医院综合改革稳步推进。创新农村经营体制机制，农村土地流转15.7万亩，农村集体“三资”委托监管代理覆盖面达100%，茶店镇“四化同步”试点、宝丰镇全国小城镇发展改革试点有序推进。严格清理核定审批事项，新上工业项目“零收费”。推进行政审批“三集中、三到位”和“四减五制六公开”，审批时间明显缩短。

　　--招商引资和对外开放再创佳绩。筹办和参与了饮水思源光彩十堰行、华创会、台湾周、京港合作等招商活动。引进市外境内项目538个，到位资金260亿元，分别增长45%、52.7%。万达集团、银轮股份、桑普太阳能、人福药业、苏酒集团、稻花香集团、国家木塑专委会等一批知名企业投资十堰。对口协作方案获国务院批复并取得明显成果，与中关村管委会签订战略合作协议。着力推进“大通关”建设，十堰出入境检验检疫局挂牌开检，新增出口企业19家，双星东风、平云工贸、东风三立车灯等进入“外贸出口A类企业”，外贸出口创历史新高。

　　（四）致力于改善民生，社会事业蓬勃发展

　　--民生福祉持续提升。坚持民生优先，加大民生投入，切实为人民群众办实事、办好事。民生支出达183.5亿元，占地方公共财政预算支出80.3%。城镇居民可支配收入和农民人均纯收入分别增加1989元、599元。新增城镇就业3.7万人。五项社会保险参保达206.4万人次。发放城乡低保资金4.4亿元，城乡低保标准分别提高23%和26%；发放救灾资金5300万元，发放老龄补贴1317.6万元；新建续建社会福利服务项目13个，改扩建农村福利院20所；发放农村五保供养资金6800万元，做到了“应保尽保、按标施保”。建设保障性住房18052套，竣工8500套，分配入住7305套。新增1对武汉至十堰动车，十堰至武当山城际公交开通运营。十件实事年度任务全面完成。

　　--社会事业全面进步。国家科技成果转化服务（十堰）示范基地、十堰商用车及关键零部件产业集群、西城省级高新区分别获科技部和省政府批准，新增高新技术企业30家，省级创新平台6家，实现高新技术产业增加值120亿元，增长20.6%。我市及张湾区、茅箭区、竹溪县分别荣获全国科技进步先进市和先进县（区）称号。优先发展教育事业，丹江口市、郧县、张湾区、茅箭区分别顺利通过国家和省义务教育均衡发展评估；学前教育三年行动计划全面完成，新建改扩建幼儿园221所；城区开工建设5所中小学，新建标准化校舍21.8万平方米，竹山一中迁建主体工程完工。老干部活动中心建成，传媒大厦、广电大厦开工建设。新增全国重点文物保护单位6家，新建农民体育健身工程100个，房县荣获全国群众体育先进县。市档案馆被评为国家一级档案馆。发放医疗救助资金7800万元，新农合参合率达98.7%，新创建省级临床重点专科11个，新增床位1418张，郧西、房县分别成功创建国家和省卫生县城。人口计生工作受到省委、省政府表彰。

　　（五）致力于生态建设，环境质量继续改善

　　--生态建设全面加强。编制完成十堰城市风貌特色规划和永久保留山体规划。通过省级森林城市创建，获准创建国家森林城市。全面推进创模“十大工程”建设，国家环保模范城市创建达标率达70%。竹溪十八里长峡晋升为国家级自然保护区。移土培肥工程基本完工。五条河治理完成示范段建设。治理水土流失面积185平方公里，人工造林31.6万亩，完成主要交通干线、城区、产业园区生态修复8000亩。我市被纳入第六批全国生态文明建设试点城市，张湾区被授予“全国国土资源节约集约模范区”，房县、丹江口市分别创建国家和省级园林城市通过验收。

　　--环境保护更加有力。实施环境保护“一票否决”制，大力开展“清水行动”。节能减排成效明显，二氧化碳排放下降3.5%。实施主要污染物总量减排项目76个，4个水泥厂提前一年完成脱硝减排工程，机动车排气监测和标识管理全面启动。工业节能增效明显，规模以上工业企业单位增加值能耗下降17%，度电产值增长16.5%。丹江口库区库底清理工作全面完成。汉江干流十堰段、丹江口水库水质达到国家地表水Ⅱ类标准，集中式饮用水源地水质达标率100%。全市空气质量优良天数达320天。

　　（六）致力于创新社会治理，民主法治建设成果丰硕

　　--民主法治建设稳步推进。认真贯彻落实中央“八项规定”，狠刹“四风”，“三公”经费明显下降。严肃查处违法违纪案件，扎实开展民主评议政风行风和“电视问政”活动。密切联系群众，推进工作落实，政府自身建设得到加强。加强法治建设，办理法律援助案件1743件，调处矛盾纠纷1.9万起，被授予“全国法治城市创建先进单位”称号。全国城市文明程度指数暗访测评取得良好成绩。高水平承办了全国创建星级文明户工作座谈会，创建文明新村357个，“十星级文明户”创建基本实现全覆盖，涌现了全国人民满意的公务员张吉来、“楚天园丁奖”获得者伍昌云等一批先进典型。自觉接受人大、政协监督，定期向人大报告工作、向政协通报情况，认真办理人大代表专项议案，城区外来务工人员生产生活得到较好保障；办理人大代表意见建议150件、代表走访选民建议108件、政协委员提案292件，办结率、满意和基本满意率均达100%。努力扩大基层民主，行政村全部实行村务公开。国防动员、民兵预备役和拥军优属工作得到加强。强化审计监督，项目管理水平和财政资金使用效益逐步提高。

　　--社会治理不断加强。社会管理综合信息平台和指挥中心建成运行，城区网格实现全覆盖，城市管理与社会综合治理网格实现无缝对接，群众安全感和治安满意度位居全省前列，竹山县荣获全国社会管理综合治理先进集体称号。大力化解信访积案，按期办结率达98%。加强食品药品和特种设备安全监管，扎实开展安全隐患排查整治，安全生产总体形势稳定向好。

　　在经济社会发展的同时，全市民族宗教、外事、侨务、对台、气象、地震、人防、残联、老龄、统计、社科、地方志等工作也都取得了新的成绩。

　　各位代表！激情创造梦想，实干成就未来。刚刚过去的一年，我市地区生产总值过千亿，人均突破5000美元大关，经济社会发展实现新跨越；有序推进老工业基地调整改造，加快生态滨江新区建设，转型发展迈出新步伐；加强中心城区建设和发展，支持各县（市）奋力崛起，“一核多支点”建设开创新局面；南水北调顺利通过国家终验并正式蓄水，与京津冀受水区的战略协作初现成效，成功搭建了以水为媒、共谋发展的新平台。一年的足迹不寻常，一年的变化不平凡！这充分展现出了十堰转型跨越发展的蓬勃生机和美好前景。成绩的取得，得益于市委的坚强领导，得益于市人大、市政协的有效监督，得益于社会各界的大力支持，凝聚着全市人民的智慧和心血。在此，我代表市人民政府，向所有参与、关心和支持十堰发展的同志们、朋友们，表示衷心的感谢并致以崇高的敬意！

　　在总结成绩的同时，我们必须保持清醒头脑，正视工作中的不足和发展中的差距：经济规模不大，产业层次和整体水平不高，经济增长的协调性、可持续性、抗风险性不够，自主创新能力与核心竞争力不强，城乡居民增收渠道不宽，消费需求不旺，少数政府部门和工作人员进取、服务意识不强，发展环境不优等问题依然存在，有些还比较严重。对此，我们将高度重视，找准根源，综合施策，下更大的决心、花更大的气力切实加以解决。

　　二、2014年工作安排

　　2014年是贯彻落实党的十八届三中全会精神、全面深化改革的开局之年，也是实施“十二五”规划的攻坚之年。我们面临的发展机遇前所未有：一是党的十八届三中全会掀开了全面深化改革的新篇章，一系列改革措施将密集出台，改革红利、内需潜力和创新活力叠加释放，有利于加速推进十堰转型升级。二是国家大力推进生态文明建设、新型城镇化和秦巴山片区扶贫开发，启动实施长江经济带战略，省委、省政府将汉江生态经济带纳入省级开发战略层面，着力构建“两圈两带”格局，将为十堰建设区域性中心城市提供强有力的支撑。三是随着“四规划一方案”、特别是对口协作方案的全面实施，十堰发展将获得更多外力支持和拉动。四是十堰迈入工业化中后期加速发展的“黄金时期”，东风和沃尔沃集团构建全球最大的商用车战略联盟，新商用车总部落户十堰，东风各事业板块和地方发展双轮驱动，将为我市转型跨越发展增添新的强大动力。

　　我们也必须清醒看到，今年宏观经济形势更加错综复杂，改革调整任务更加艰巨繁重，经济增长的不确定因素仍然存在，制约十堰发展的重大矛盾和瓶颈没有得到彻底解决，周边区域竞相发展带来的巨大挑战和竞争压力不容忽视。我们必须坚持底线思维，紧紧抓住全面深化改革的重大历史机遇，紧紧抓住新的发展态势和新的发展基础条件创造的有利时机，增强机遇意识、进取意识、担当意识，以更大力度攻改革之坚、克转型之难，使十堰经济更好、结构更优、质量更高、后劲更足、实力更强。

　　今年政府工作的总体要求是：以党的十八大、十八届三中全会和中央、省经济工作会议精神为指导，坚持“竞进提质、升级增效”的总要求，把改革创新贯穿于经济社会发展的各个领域、各个环节，着力激发市场活力，努力实现经济升级增效、民生不断改善、生态持续优化、调水顺利实现、社会和谐稳定，为生态文明建设和转型跨越发展走在全省前列、建设区域性中心城市奠定坚实基础。

　　经济社会发展的主要预期目标是：地区生产总值增长11%；规模以上工业增加值增长13%；固定资产投资增长25%以上；地方公共财政预算收入增长13%；社会消费品零售总额增长15%；实际利用外资增长15%；外贸出口增长12%；城镇居民人均可支配收入增长11%，农民人均纯收入增长12%；城镇登记失业率控制在4.6%以内；居民消费价格指数、单位生产总值能耗下降、主要污染物排放量控制在省定目标以内。

　　上述目标是必达目标，实际工作中我们还要努力争取实现质量更高、速度更快的增长。这是因为，发展是第一要务，是解决一切问题的关键，而十堰的主要问题是发展不够。中央强调不简单以GDP增长论英雄，我们的发展压力不是小了，而是更大了；任务不是轻了，而是更重了；要求不是低了，而是更高了。而且着眼于生态文明建设和转型跨越发展走在全省前列、建设区域性中心城市的总目标，面对加快发展、加快转型的双重任务，我们必须同时做好扩大总量和提升质量的两篇文章。底盘做不大，就成不了中心；质量提不高，就走不到前列。因此，我们务必正确处理好保持经济持续健康发展与GDP增长的关系，努力在转型发展中实现跨越发展。重点做好以下工作：

　　（一）坚定不移全面深化改革开放，着力激发市场活力

　　改革开放是当代中国发展的活力之源，是我们大踏步赶上时代前进步伐的重要法宝。要坚持以改革统领各项工作，把改革的举措与调整的举措、发展的举措有机统一起来，按照中央和省、市委的部署，抓紧研究制定推进改革的方案，不等待、不观望、不懈怠，推动重点领域和关键环节改革取得实质性进展。

　　--紧紧围绕使市场在资源配置中起决定性作用深化经济体制改革。一是建立和完善市场主体快速发展的机制。全面放开，竭力搞活，“松绑、放权、减负”，全力支持非公有制经济发展。放手发展各类市场主体，废除对非公有制经济的不合理规定，消除隐性壁垒，营造浓烈氛围，创造优越条件，促进民营经济大发展大提升，让一切市场主体的活力竞相迸发，让市场的力量充分涌流，让企业的能量尽情释放，形成推动十堰发展的强大动力。二是加快转变政府职能。处理好政府与市场的关系，全面正确履行政府职能，该管的管到位，该放的放彻底，切实把职能转向为市场主体营造良好发展环境、提供优质服务和维护社会公平正义上来，努力做到不越位、不错位、不缺位。简政放权，坚决做到“三个一律”，即市场机制能有效调节的经济活动，一律取消审批；社会组织能参与承担的事项，一律转移给社会组织；面向基层且由地方管理更方便有效的经济社会事项，一律下放县（市区）管理。同时，建立行政审批统一目录，优化再造服务流程，并向社会公布。强化效率导向，雷厉风行，急事急办、要事快办、特事特办，推进政府工作提质提速提效。三是深化财政金融体制改革。进一步研究市区财政分享办法，建立激励三区加快发展和财权事权统一的体制机制。做好财政资金统筹整合工作，提高财政资金使用效益。抓好多层次资本市场建设，稳步发展金融资产交易等要素市场，支持金融机构各类重大改革创新措施先行先试。四是深入推进社会事业领域改革。扩大基本药物制度覆盖面，50%县市启动公立医院综合改革试点工作，鼓励引导社会资本办医。完善现代公共服务体系。加快新型城镇化改革，争创全国新型城镇化试点城市。

　　--全方位扩大对外开放，着力构建开放型经济新体制。一是努力提升招商质效。坚持把招商引资作为调结构、促转型的有力抓手，围绕产业招商、主题招商、区域招商“三个重点”，坚持央企、民企、外企引进“三位一体”，突出招商引资与招商选资、招大引强与全民创业、引进资金与引进技术“三个结合”，有针对性地开展上门招商活动。深入推进京津冀对口协作，精心组织重大招商主题活动，坚持首席服务官制度，加大跟踪服务力度，促进招商引资实现量的扩张和质的提升，力争到位资金突破360亿元。二是大力发展外贸经济。积极支持东风公司、双星东风、运银实业、聚达食品等企业开拓国际市场，争创国家级出口汽车及零部件产品质量安全示范区，扩大出口规模。三是建好用好对外开放平台。加快海关监管区、公路二类口岸、国家航空一级口岸等出口平台建设，加强沿海沿边通关协作。积极做好外派劳务综合服务平台建设，大力发展外派劳务经济。

　　--大力推进科技创新，不断增强发展动力。加快十堰市秦巴山片区科技创新中心建设，建成国家汽车零部件质检中心。加快十堰经济开发区、西城高新区和十堰北京工业园科技创新产业园建设，新认定国家高新企业30家，新增省级创新平台10家，高新产业增加值占GDP的比重达到12%。

　　（二）坚定不移推进经济转型升级，着力提升发展质效

　　--调整优化结构，做强做大工业经济。坚持“效速兼取、轻重齐抓、央地并进、市县共兴”的方略，进一步强化工业兴市意识，推进工业倍增计划实施，优化提升“一主”、培育壮大“多元”、强力扶持“中小”、做精做专“特色”，构筑转型跨越坚实支撑，不断增强工业核心竞争力，力争实现规模以上工业企业总产值2000亿元、增加值580亿元，新增规模以上工业企业150家。一是做强支柱产业。巩固汽车产业的龙头地位，全力服务东风公司与沃尔沃战略合作和各事业板块在十堰的发展，推进十堰新基地项目建设，发展地方汽车及零部件产业，争取早日实现产值过1500亿元。加快发展农产品加工、生物医药、食品饮料、新型建筑材料、新能源等新兴产业 ，形成若干过百亿元的产业集群，力争生物产业跨入省级重点集群行列，构建一主多元工业产业格局。二是培植骨干企业。大力支持和培植一批投入产出过百亿、十亿元的重点骨干企业，全力提供要素保障，及时帮助解决困难。深入实施中小企业成长工程，启动千家企业培育计划，着力扶持小微企业发展壮大，形成引领作用明显、支撑地位突出、后续力量强劲的庞大企业群体。三是推进产业园区建设。进一步配套完善基础设施，加快城区生物产业园、新型建材园、农产品加工园等园区建设。支持十堰开发区拓展空间，推进国家高新技术开发区创建，加快各县市特色园区建设，为工业发展提供足够的空间支撑。推动“满园工程”和工业项目建设，开工建设东风零部件工业园、特种商用车等一批新项目，推进东风动力总成二期、装备工业园二期、东风小康30万台发动机等一批在建项目早日竣工投产。四是进一步加大培育企业家的力度。切实维护企业家合法权益，营造企业家健康成长的浓厚氛围。加强企业家互动交流和学习培训，提升现代企业经营管理理念。建立健全企业家信用评级机制，鼓励诚信经营、依法经营、回报社会，为社会创造更多财富和就业机会。

　　--坚持强农富农美农，加快发展现代农业。强化农业重中之重地位，增强“首位度”意识，着眼于“农业强、农民富、农村美”，以产业化为方向，围绕“基地做大、龙头做强、产品做优、品牌做响”四大重点，开展精深加工，推进深度开发，推动“三农”工作全面进步。一是提高农业产业化水平。在“种、养、加、销”产业链各环节建设上打组合拳，见连环效。稳定粮食生产，确保总产22亿斤以上。深入推进“四百万”特色产业基地建设，新建茶叶、中药材、核桃基地30万亩，新增山羊出栏6万只。大力发展林业经济，提高林业对农村经济的贡献率。进一步把农产品加工作为农业产业化的牛鼻子抓实抓好，每个县（市）都要建设农产品加工园，积极引进和培植壮大龙头企业，新增亿元以上的农产品加工企业10家以上，力争全市农产品加工产值达到280亿元。着力拓展农产品营销渠道，提高市场化水平。推进农业生产加工标准化，加大品牌建设力度，提高农产品质量，保障舌尖上的安全，新增“三品一标”品牌认证10个以上。二是创新农村经营机制。推进家庭经营、集体经营、合作经营、企业经营等方式创新，让农村土地流转起来，资产经营起来，农民组织起来。鼓励业主进村、推动土地（山场）资源流转，新培育发展家庭农场100家、专业大户1000 家，农民专业合作社100家，土地流转15万亩。三是加强基础设施建设。加大农田水利、农村道路、农村环境综合整治等建设力度，增强农业抗御自然灾害能力，提升产出水平。改造县乡道58公里，新建通村水泥路800公里；除险加固病险水库41座，解决12万人农村饮水安全问题。以丹江口库区和中心城区周边50个村为试点，深入实施宜居住房、清洁生产、安全饮水、通畅路网、环境治理、乡村绿化六大工程，大力推进美丽乡村建设。四是推进农业科技进步。促进农业科技深度融合，加快农业科技成果推广应用，提高农民科技转化素质，培养新型职业农民。

　　--培育新兴业态，促进第三产业提档升级。充分发挥现代服务业在优化产业结构，增强城市吸引力、辐射力和现代化水平中的引擎作用，围绕发展生产、改善生活和扩大就业，加快各类服务业发展，实现比重提高、水平提升、竞争力增强。突出发展现代服务业，形成第三产业新的强势增长点。实现增加值430亿元以上，新增限上企业150家，新增贷款150亿元以上。一是做强旅游产业。在抓好武当山发展的同时，集中精力推进城区城郊生态游憩带建设，着力打造特色小镇、旅游名村，开辟我市旅游的新空间新业态，为市民建好休闲度假、放松身心的好去处。支持丹江大坝、小太平洋等景区创建5A级旅游区，武当·太极湖创建国家级旅游度假区。打造饮水之源·感恩之旅精品旅游线路，培育“大十堰”旅游套餐。力争全年接待游客3300万人次，实现旅游收入230亿元，增幅15%以上。二是做大商贸物流业。紧盯把物流业培植成新的支柱产业的目标，服务好林安、昌升国际、寿康、新合作、汉江等物流项目建设，开工建设万达广场、华西国际农商城，启动国家级农产品现代流通综合试点。继续实施万村千乡市场工程，突出抓好城乡流通网络和信息化建设，新建和改扩建各类市场100个、便民超市200个，培育过亿元商贸物流市场16个，加快“武当珍品汇”在全国布点。三是做优新兴服务业。大力发展健康、养老、家政、电商、信息、金融保险等现代服务业，促进健康业、养老业取得明显进展，争取立项建设汽车整车及零部件交易所、电子商务产业园区。

　　--持续扩大投资，增强发展后劲。坚持把项目和投资作为经济增长的发动机和大底盘，充分发挥牵引和承载作用。一是千方百计争取项目。坚持向政策和市场要项目，努力扩大投资渠道。捕捉投资信息，抢抓政策机遇，大力争取国家投资，确保中央和省投资明显高于上年。创新信用环境，改善金融服务，争取金融对十堰发展更多支持。充分利用优势资源、特色产业、知名品牌，吸引外来投资，激活民间投资，确保全社会固定资产投资突破1100亿元。二是全力以赴建设项目。完善领导包保、部门服务、专班推进的工作机制，促进多上项目，上大项目。突出抓好重大基础设施和生产性项目建设，加快武当山机场建设进度，力争主体工程基本完工；谷竹高速全线通车，郧十、十房高速主体工程完工；做好武襄十城际铁路、十巫高速等项目前期工作；郧县长沙一级路、郧西土门至天丰一级路、方辽路、十竹路建成通车，改造升级346国道和316国道白浪段、黄龙至柏林段，加快环丹江口库区公路建设；推进京能热电、夹河关电站、孤山电站建设，龙背湾电站实现并网发电。三是精心策划储备项目。研究国家政策走向，紧盯国家投资重点，做深项目前期工作，着重在装备制造、高新技术、现代物流、健康养老、生态建设等领域，策划包装一批投资规模大、带动作用强的重大项目，充实完善项目库，确保项目储备充实、接替有序、支撑有力。

　　（三）坚定不移构建“一核多支点”，着力加快区域统筹协调发展

　　城镇化是现代化的必由之路，是推动区域协调发展的有力支撑。坚持以推进新型城镇化统领城乡发展，城镇化率提高2%。继续做到精心规划、精致建设、精细管理，本着对历史对人民高度负责的精神，切实提高城市建设水平，把“让群众生活更舒适”这一理念融入城市规划建设管理的血脉里。

　　--大力加强中心城区建设。不断完善基础设施，提升城市综合功能。新建林荫大道二期、风神大道二期、发展大道延长线、北环路一期、机场东路、湖南路、清潭路，续建西城大道、郧阳路、京东路，改造大岭路、镜潭路、武当路、襄阳路、源园路和22条背街小巷，打通10处市政道路微循环。加快建设火车站北广场、生态滨江新区重点路网、互联网、电讯网，支持湖北医药学院长岭校区、市人民医院滨江医院建设。全面加强城中村改制改造、棚户区改造工作。继续深入开展“三违”治理和市容环境综合整治，加快数字化城管指挥中心建设，推进城市管理的规范化、常态化、标准化。

　　--强力发展县域经济。把发展县域经济作为战略性工程来抓，进一步增强动力、激发活力、提升能力，营造县（市）之间彰显特色、争先进位、竞相发展的生动局面。切实支持丹江“中国水都、十堰龙头”、郧县“生态滨江新区”、房县“四化”协调发展示范区、郧西“文化旅游强县、十堰西北门户”、竹山“十星高地、秦巴强县”，竹溪“绿色崛起示范县”建设，纵深推进竹房城镇带建设，加快汉江生态经济带综合开发步伐。重点加快房县北城生态工业园、竹山宝丰创业园、竹溪人福生物产业园、郧县杨溪生态产业园、郧西七夕文化产业园等园区建设，启动丹江口库区高效生态农业示范区建设，不断提高县域经济比重，壮大县域综合实力。

　　--深入推进扶贫开发。抢抓秦巴山片区扶贫攻坚政策机遇，强力推进基础设施、产业扶贫、生态环保、民生改善、科技扶贫五大攻坚。启动65个重点贫困村整村推进，推进柏林片区、大川片区为重点的城区农村扶贫开发，完成扶贫搬迁3900户，培育扶贫龙头企业30家，力争全年减少贫困人口9万人。

　　（四）坚定不移加强生态文明建设，着力创建国家生态文明示范区

　　牢固确立生态立市意识，牢固树立尊重自然、顺应自然、保护自然的生态文明理念，把生态文明建设融入经济、政治、文化、社会建设各方面和全过程。既要金山银山，更要绿水青山，推进绿色发展、生态崛起，使生态成为十堰发展的主旋律，绿色成为十堰发展的主色调，培植十堰生态优势，铸造十堰生态品牌，努力建设天蓝地绿、气清水碧的美丽十堰。

　　--加快重点生态工程建设。实施水土保持重点防治工程，开展“绿满十堰”行动，推进四方山森林公园、岩洞沟水库生态区建设。突出抓好交通干道沿线、产业园区、“口子乡镇”等重点区域造林绿化和生态修复，加强裸露山体治理，建立重点区域生态修复长效机制。加强森林资源保护，提高森林覆盖率。抓好新一轮退耕还林工程建设，加快丹江口库岸和汉江、堵河两岸绿化工程建设，完成人工造林30万亩、义务植树1000万株。抓好饮用水水源保护，保障饮水安全。

　　--加强节能减排和环境保护。推进循环经济和清洁生产，加强主要污染物总量减排工作，控制机动车氮氧化物排放。开展城区大气污染防治，加强渣土运输、施工场地等扬尘监管。加大水污染防治和水土保持规划实施的监管和考核力度，推进农村面源污染综合治理，深入开展“清水行动”，健全环境执法长效机制。全面启动神定河、泗河、犟河、剑河、官山河等流域生态景观建设和综合治理，推进污水处理厂改制和运营监管到位，再建城市污水管网150公里，整治排污口100个，建设神定河污水处理厂提标扩能项目，确保南水北调中线工程调水如期进行。

　　--深入推进生态文明创建。加强生态文明制度建设，实行最严格的耕地保护、水资源管理等制度，探索建立有利于生态文明建设的政绩考核评价体系。全力推进“五城联创”，积极争取将十堰纳入国家生态文明先行示范区试点范围。深入推进创模“十大工程”建设，全面开展“六大绿色创建”，确保2014年完成90%创模指标任务和创建全国文明城市、国家森林城市年度任务，继续巩固创卫成果。积极推进野人谷晋升国家级自然保护区、黄龙滩晋升国家级湿地公园、大西沟晋升省级自然保护区等工作。

　　（五）坚定不移保障和改善民生，着力建设幸福十堰

　　突出执政为民，坚持把保障和改善民生作为一切工作的根本出发点和落脚点，尽心竭力解决民生问题，建设幸福十堰。

　　--推进社会事业均衡发展。优化城乡资源配置，大力促进教育公平，健全学生资助体系，加快农村寄宿制学校配套设施建设。推进车城高中、十堰高级职业学校、柳林中学、重庆路小学整体迁建，加快北京路中学、龙门小学建设，确保武当山国际武术学院秋季开学，大力支持高校发展。健全完善突发公共卫生事件应急和重大疾病防控机制，应急处置率达100%；健全新农合制度，确保参合率稳定在98%以上；继续提高医疗救助住院平均核销比例，完善临时救助制度；大力开展“健康城市”创建活动，免费向城乡居民提供11类公共卫生服务，加快村卫生室标准化建设。继续稳定低生育水平，综合治理出生人口性别比，提升计生优质服务水平。

　　--提高就业和社会保障水平。实施创业促就业工程，完善城乡均等的就业创业服务体系，城镇新增就业5.3万人。扩大社会保险覆盖面，五项社会保险参保人数达到207万人。大力实施“3331人才成长工程”，新增专业技术人才5000人、高技能人才4500人。推进养老机构建设，新建1所县级社会福利服务中心，改扩建18所农村福利院。启动实施南水北调移民安稳致富工程，全面做好移民后期扶持工作，把移民安置点建成新农村示范区。加大住房保障力度，进一步扩大公积金受益人群。拓宽城乡居民增收渠道，让发展成果更多更公平惠及全市人民。

　　--创新发展文化事业和文化产业。坚持公益性文化事业和经营性文化产业两手抓，构建现代公共文化服务体系。加快南水北调博物馆建设。深度挖掘地方传统文化，大力创作文艺精品，积极推动文艺产品市场化、产业化运作。大力实施文化项目招商，重点培育影视制作、时尚设计、动漫游戏等文化产业，力争增加值增长20%。加快发展体育事业，新建农民体育健身工程100个、健身路径50条，开工建设城区体育公园。

　　（六）坚定不移推进法治十堰建设，着力提升社会治理水平

　　--创新社会治理机制。坚持源头治理、综合治理、系统治理，提升网格化管理、社会化服务水平。深化平安十堰创建，大力实施“天网工程”，构建立体防控体系；创新预防和化解社会矛盾机制，有效化解社会矛盾；加快户籍制度改革，强化流动人口和特殊人群管理服务。强化检验检测和日常监管，提高食品药品和特种设备安全保障水平。深入推进质量兴市、品牌强市，健全消费者权益保护体系，保障消费安全。落实最严格的安全生产责任制，坚决防范重特大事故发生。认真做好群众来信来访工作，加强城市物业、供热和车辆停放等管理，大力解决群众反映的热点难点问题。构建科学有效双拥优抚安置体系，巩固双拥共建、军民团结良好局面。

　　--推进民主法治十堰建设。坚持依法行政，畅通民主渠道，扩大政务公开，自觉接受人大法律监督和政协民主监督，加强与各民主党派、工商联、人民团体的联系，主动接受新闻媒体和社会监督，建立健全科学民主决策机制，努力在公众监督下把政府工作做得更好。继续开展全国村务公开民主管理示范单位与和谐社区示范单位创建活动。深入推进法治政府建设，增强依法行政能力。认真落实“六五”普法规划和《法治十堰建设纲要》，不断提高全社会法治化管理水平。

　　--深入开展精神文明创建。努力做好全国、省级文明城市综合验收考评工作。全面推进“十星级”系列创建，扎实抓好各行业道德讲堂建设，大力开展道德模范、身边好人学习宣传教育等活动。广泛开展志愿服务活动，大力实施文明交通、文明旅游、文明网络、文明餐桌四大行动。深化精神文明创建活动，不断提高文明素养，培育良好社会风尚，凝聚建设和谐十堰的正能量。

　　（七）坚定不移提升工作效能，着力建设人民满意政府

　　各级政府和政府工作人员是受人民之托，为人民履职。全心全意为人民服务，是我们的立政之本；人民对美好生活的向往，是我们的奋斗目标。我们时刻牢记并切实做到“善政、勤政、明政、严政、廉政、实政”，让人民群众满意。始终保持强烈的使命感、责任感、紧迫感，始终保持昂扬锐气、充盈底气、蓬勃朝气，把精神状态调到最佳，把工作标准调到最高，把工作干劲调到最大，把工作作风调到最实，以对人民高度负责的态度，不为任何困难所阻，不因任何干扰所惑，冲在前线，战在前方，大力破解工作难题，切实提高工作成效。以开展群众路线教育实践活动为切入点，自觉践行为民、务实、清廉，把人民利益摆在最高位置，把群众冷暖时刻挂在心上，树立群众观点，把握群众脉搏，倾听群众呼声，解决群众诉求，增进群众感情，从群众最不满意的事情抓起，从群众最困难的事情帮起，从群众最期盼的事情做起，做到民有所呼我有所应，民有所求我有所为，民有所盼我有所办，用我们的辛苦指数提升人民群众的幸福指数。

　　我们时刻牢记并切实做到坚持求真务实，狠抓落实。组织任何工作都要身体力行，率先垂范，少讲空话，多干实事。干任何事情都要突出“实”字，深入实际，了解实情，多谋善干、善谋实干，力戒形式主义和官僚主义，防止急功近利，不搞形象工程，多做打基础、添后劲、利长远的工作，多办顺民意、惠民生、得民心的实事，不搞短期行为，不玩数字游戏，不留发展后患，拼出没有水分的增长、实实在在的发展，做出无愧于历史、无愧于时代、无愧于人民的业绩。要脚踏实地干好工作，在狠抓落实上下足功夫，解放思想抓落实，转变作风抓落实，突出重点抓落实，明确职责抓落实，强化督办抓落实，精心部署点点入地，真抓实干步步夯实。讲究抓落实的艺术，提高抓落实的本领，改进抓落实的方法，切实做到目标坚定不移、精力全神贯注、措施扎实具体、执行坚决有力、结果富有成效。

　　我们时刻牢记并切实做到立身不忘做人之本，为政不移公仆之心，用权不谋一己之私。发扬艰苦朴素的优良传统，坚决制止铺张浪费的奢侈行为，自觉遵守八项规定，切实做到廉洁从政。推进行政权力运行公开透明，加强项目、资金、工程等重点领域的监管和审计工作，建立健全土地出让、工程建设、政府采购等重点领域公正透明交易平台，实行阳光操作。深入开展百姓问政、网络问政和民主评议政风行风活动，坚持用制度管权管事管人，严格遵守廉政准则，认真履行“一岗双责”，全面落实党风廉政建设责任制，加强公务员队伍教育监督和管理，严肃查处各类腐败行为，以廉政建设的实际成果取信于民。

　　各位代表！为人民群众办好事、办实事是政府应尽的职责。今年将继续办好十件实事：一是建设标准化村卫生室140个；二是建设社区居家养老服务中心60个；三是开通城区6条通乡（镇村）公交；四是创建生态乡镇25个、生态村300个；五是改造微循环道路10条、背街小巷22条；六是新建市民健身苑10个；七是开工建设保障性住房3万套；八是建设“平价菜店”10家；九是对65个扶贫重点村实施整村推进；十是完成城区中小学D级危房改造3万平米，启动重庆路小学、车城高中迁建。

　　各位代表！百舸争流，破浪者领航；千帆竞发，奋勇者当先！让我们团结起来，在市委的坚强领导下，胸怀跨越梦想，勇担历史重任，永葆不懈斗志，大兴实干之风，奋力谱写十堰生态文明建设和转型跨越发展走在全省前列、建设区域性中心城市的辉煌篇章！
